# The physician as a success determining factor in CT-guided pain therapy

**DOI:** 10.1186/s12880-020-00544-6

**Published:** 2021-01-13

**Authors:** Christoph A. Stueckle, Benedikt Hackert, Sarah Talarczyk, Martin Wawro, Patrick Haage, Ulrich Weger

**Affiliations:** 1 Faculty of Health, Witten/Herdecke University, Witten, Germany; 2 Department of Diagnostic and Interventional Radiology, HELIOS University Hospital Wuppertal, University Witten/Herdecke, Wuppertal, Germany; 3 Department of Psychology, Faculty of Health, Witten/Herdecke University, Witten, Germany; 4 MVZ Professor Uhlenbrock Und Partner GmbH, Dortmund, Germany

**Keywords:** CT guided therapy, Back pain, Periradicular therapy, Psychological cofactors, Expectation effects, Physician–patient relationship

## Abstract

**Background:**

Back pain is a common problem and a burden for the patient. MR-morphologically proven pain-causing changes of the spine is often successfully treated utilizing CT-guided pain therapy. The CT-guided execution enables a controlled and reproducible therapy. Nevertheless, treatment results can differ even with the same patient; the physician is a possible influencing factor of the outcome. Accordingly, the present study analyzes the different behaviors and forms of communication of the treating physicians during the course of the intervention as factors influencing the outcome of treatment.

**Methods:**

67 patients suffering from specific back pain were included in this study. 5 treating physicians (2 female, 3 male) of different age (29–63 years), and experience and a total of 244 CT-guided treatments were included in this study. In every case a psychologist observed the treatment based on a standardized observation protocol. Observed were both the verbal and non-verbal interactions as well as the reaction of patient and physician. The success of the therapy was measured in the course of the treatment using the visual analogue pain scale. The technical comparability of the performed CT-guided periradicular therapy was ensured by the distribution of the drug mixture.

**Results:**

The outcome is significantly better if the patient considers the treating physician to be competent (correlation coefficient: 0.24, *p* < 0.006) and feels understood (correlation coefficient: 0.29, *p* < 0.001). In addition, the outcome is better when the physician believes that the treatment brings a positive reduction of pain, underlining his belief with positive statements of affirmation before the intervention thus creating a positive atmosphere [correlation coefficient: 0.24 (*p* < 0.009)]. In contrast, the outcome is worse if the patient complains about pain during the intervention [average pain reduction M = 0.9 (pain group) vs. M = 2.0 (no-pain group)].

**Conclusion:**

Our study shows that with comparable implementation of CT-guided periradicular therapy, the outcome of the patient with specific back pain can be significantly improved by certain behavioral patterns of the performing physician and this without side effects and without significant additional time expenditure. Our findings indicate that there is a non-negligible psychological factor linking confidence in therapy to actual therapy success.

*Trial Registration*: The study was designed as an observational study, therefore a trial registration was not necessary.

## Background

Pain, especially lower back pain, is a common problem in the industrialized world with manifold reasons. The reported lifetime prevalence varies from 20 to 40% and causes annual costs in the US of $87.6 billion in 2013 [1–3]. Research of pain treatment shows a positive correlation between pain reduction and quality of life [4]. There are different forms of therapies that seek to reintegrate patients with chronic back pain into their life and work environment. Therapies comprise different approaches, including physical exercise, physical rehabilitation, acupuncture, pharmacologic management, psychological intervention, CT guided minimal invasive pain therapy and surgical intervention as well as combinations of the above mentioned [5]. Recently multi-modality therapy to treat patients with chronic back pain has become popular. Multi-modality therapy combines different treatment approaches to cover a wide therapeutic base in a patient-centered setting [6]. This kind of therapy shows positive effects, but is at the same time very cost intensive [7].

Hence, there is a strong economic need to reevaluate the different forms of therapy in order to determine which therapy is most beneficial for the patient while considering the economic impact. This need is exacerbated by another major problem concerning the availability of the different therapy forms in the industrialized world. The limited availability of resources, combined with the cost of treatment leads to a restriction of offered therapy modalities [7]. For example, in Germany patients have to be seen by a physician specialized in pain therapy before a CT guided pain therapy can be administered. This leads to a selection of suitable patients for different therapy forms. One criterion for selection is the classification for specific or non-specific back pain. In cases of specific back pain there is an identifiable morphologic cause for the reported pain whereas in non-specific back pain such a cause is absent [8, 9].

In the present study we focus only on specific back pain with proven morphological causes for the correlated back pain.

It would be desirable to find a treatment or combination of treatment forms that show the best outcome at low risk and low cost. The different methods of treatment and the combination of them lead to a better therapy outcome, but the most relevant predictors of patient benefits remain unclear. In this regard, there is another important issue in research concerning the treatment of back pain, namely psychological effects on pain improvement originating from the doctor–patient relationship [10, 11]. The doctor–patient relationship and the interaction between them exist in nearly every kind of treatment. This is relevant in finding the most efficient treatment option, since changes in the physician's behavior could lead to an improvement in patients’ outcome in any therapy option where patient and physician interact. Hence, specific behavioral approaches to communicate with the patient during treatment, both verbal and non-verbal, should be evaluated with respect to their impact on pain reduction [12].

CT-guided pain intervention provides a high level of safety and accuracy in back pain treatment. Success rates span from 52 to 90%. Since CT-guided pain therapy should ensure comparable results as far as possible independently of the physician performing it, a physician-related success component may be assumed. [13–15].

The purpose of the presented study is to examine which factors in doctor-patient interaction lead to an improved outcome in CT guided periradicular therapy under the assumption that the technical performance of CT guided periradicular therapy is comparable.

## Methods

The presented study was approved by the local ethics committee. From 11/2016 to 7/2018 we examined a total of 354 patients with specific back pain. A pain physician admitted all patients to the CT guided interventional therapy. To execute the interventional procedure, one physician was randomly selected from a group of five radiologists (3 male, 2 female) whose experience levels ranged from 2 to 18 years (Table 1). All physicians followed the same intervention protocol. Every intervention was retrospectively reviewed by a senior expert to ensure that the intervention met the required quality criteria. All patient included in the study gave written consent before participating.

**Table 1:** Characteristics of the treating physicians

Gender	f	f	m	m	m
Age	62	28	44	40	39
CT guided Interventions done before	> 1000	≤ 500	> 1000	> 500 ≤ 1000	> 500 ≤ 1000

From all examined patients, 67 patients (30 male, 37 female) with a total of 244 examinations fulfilled the inclusion criteria and consented to participate in the study (Table 2). After an MRI scan of the back in which the morphologic reason for the back pain was identified, the patient was initially seen by a psychologist who conducted a structured pain interview, including aspects of pain duration, type of pain and any previous treatments. Afterward the patient received an explanation from the radiologist on the examination results, information on the planned procedure, advantages, potential side effects, and chances of success. Only those patients with MR pathology corresponding to the complaint symptoms were considered in the present study. The MR morphological changes were evaluated before the examination to appropriately plan the therapy. For better clarity and comparability, retrospectively assigned to the group of cases with and without pain reduction under therapy (Tables 3, 4).

**Table 2:** Characteristics of the patients

	Male	Female	Total
Number of patients	30	37	67
Examinations total	112	132	244
Average pain score before treatment	7.3	7.1	7.2
Standard deviation of pain score before treatment	1.59	2.02	1.83
Median of pain score before treatment	7	8	7
Range of pain score before treatment	4–10	3–10	3–10
Average pain score after treatment	4.2	4.1	4.1
Standard deviation of pain score after treatment	1.91	2.22	2.1
Median of pain score after treatment	4	4	4
Range of pain score before treatment	0–9	0–8	0–9

**Table 3:** Characteristics of patients divided into cases of improvement or non-improvement

	Improvement	No improvement
Average degree of osteochondrosis (0: no signs, 1: mild to 3: severe)	1.31	1.09
Average degree of spinal stenosis (%)	22.3	20.8
Nerve affection (%)	74	77
Average degree of facet arthrosis (0: no signs, 1: mild to 3: severe)	1.3	1.5
Nerve compression (%)	26	23
Cervical intervention	29	26
Lumbal intervention	71	74

**Table 4:** Comparable MR findings in both groups

	Improvement	No improvement
Average age	55.3	54.7
Female (%)	54	56
Male (%)	46	44
BMI	28.6	27.4
Diabetes (%)	8.7	11.1
Hypertension (%)	37.7	33.3
German nationality (%)	80	78
Turkish nationality (%)	9	11
Persian nationality (%)	2	0
Polish nationality (%)	9	11

The statistical analysis of the mean values shows no significant deviation between the two groups

Immediately following the consultation with the radiologist, the psychologist again saw the patient, conducting another structured query concerning the patient’s expectations of the treatment and impressions about the radiologist, which included:

- expectation regarding (further) improvement of the pain.
- likelihood that the pain is successfully treated with the procedure(s) and will disappear.
- competency of the treating physician.
- the extent to which the patient feels understood by the radiologist regarding his/her condition.

In addition, the radiologist also filled out a questionnaire querying his opinion on the chance of success for the individual patient.

After the initial examinations, the patients followed up on the CT-guided periradicular therapy. In 31% of the patients the intervention was performed on the cervical spine and in 69% of patients on the lumbar spine. In this type of procedure, a needle is positioned directly at the neuroforamen under CT control and, after verification of the needle position by means of contrast medium injection, the drug mixture of local anesthetic and glucocorticoid is administered. We used triamcinolone in a uniform dosage of 20 mg (20 mg per ml, Triam®, Winthrop Arzneimittel GmbH, Mühlheim, Germany) in each patient. As a local anesthetic we applied 2% mepivacain (20 mg/ml, Actavis-Mepivacain, Actavis Group, Hafnarfridi, Iceland) in an amount of 2 ml in each patient. For the documentation of drug distribution 1 ml of iodine-based contrast agent (Accupaqe 240, GE Healthcare, Munich, Germany) was injected.

The time frame after the initial examination varied from 1 to 14 days (average: 5 days). Prior to every treatment session, the patient was seen by the psychologist, receiving another structured query about his/her current well-being, and a re-evaluation of the current pain score. The psychologist, who observed the atmosphere and the interaction of the doctor and patient during the treatment, documented the CT-guided pain therapy treatment on a structured chart.

Following each procedure, the patient was re-interviewed by the psychologist querying:

- current pain level on a 0–10 continuous pain scale,
- expectation of (further) improvement,
- competency of the treating physician and evaluation of the doctor–patient relationship.

The names of the physicians were omitted from all patient questionnaires to prevent any impact on treatment behavior.

In order to categorize the success of the treatment, we chose to monitor the decrease in pain levels of the patients. We classified pain reduction scores of less than 0.25 as unsuccessful, with scores of 0.25–3.0 as good success and all decreases bigger than 3.0 as excellent success.

Four to 12 weeks after the end of the treatment we invited the patients to a final conversation or telephone interview to examine the long-term effects of the therapy.

For statistical evaluation of the technical and non-technical data, a database was set up that was subject to computer-assisted evaluation using MatLab(R) R2017b. Depending on the evaluation criteria, subgroups were formed where all relevant data points were available. We defined all results with a *p *≤ 0.05 as significant. In assessing the strength of the effect of correlation, we followed a modified form of the Cohen’s classification, in which a classification into relatively small, typical, and relatively large is proposed [16].

## Results

In 209 cases (86%) an improvement after treatment was reported. Most patients showed a good reduction of pain (53%) whereas 33% exhibited an excellent pain reduction.

In 35 cases (14%) no improvement was documented. To evaluate if a potential dependency on an individual physician affects the general improvement of a patient, an exact Fisher test was performed; the null hypothesis was set to indicate that improvement does not depend on the treating physician. The null hypothesis was rejected with *p* < 0.0084 on the general improvement (n = 209). Therefore, there is a dependency on how the physician behaves or how the physician interacts with the patient in respect to therapy outcome. No significant statistical correlation between age, gender and experience of the physician could be demonstrated.

**Fig. 1:**
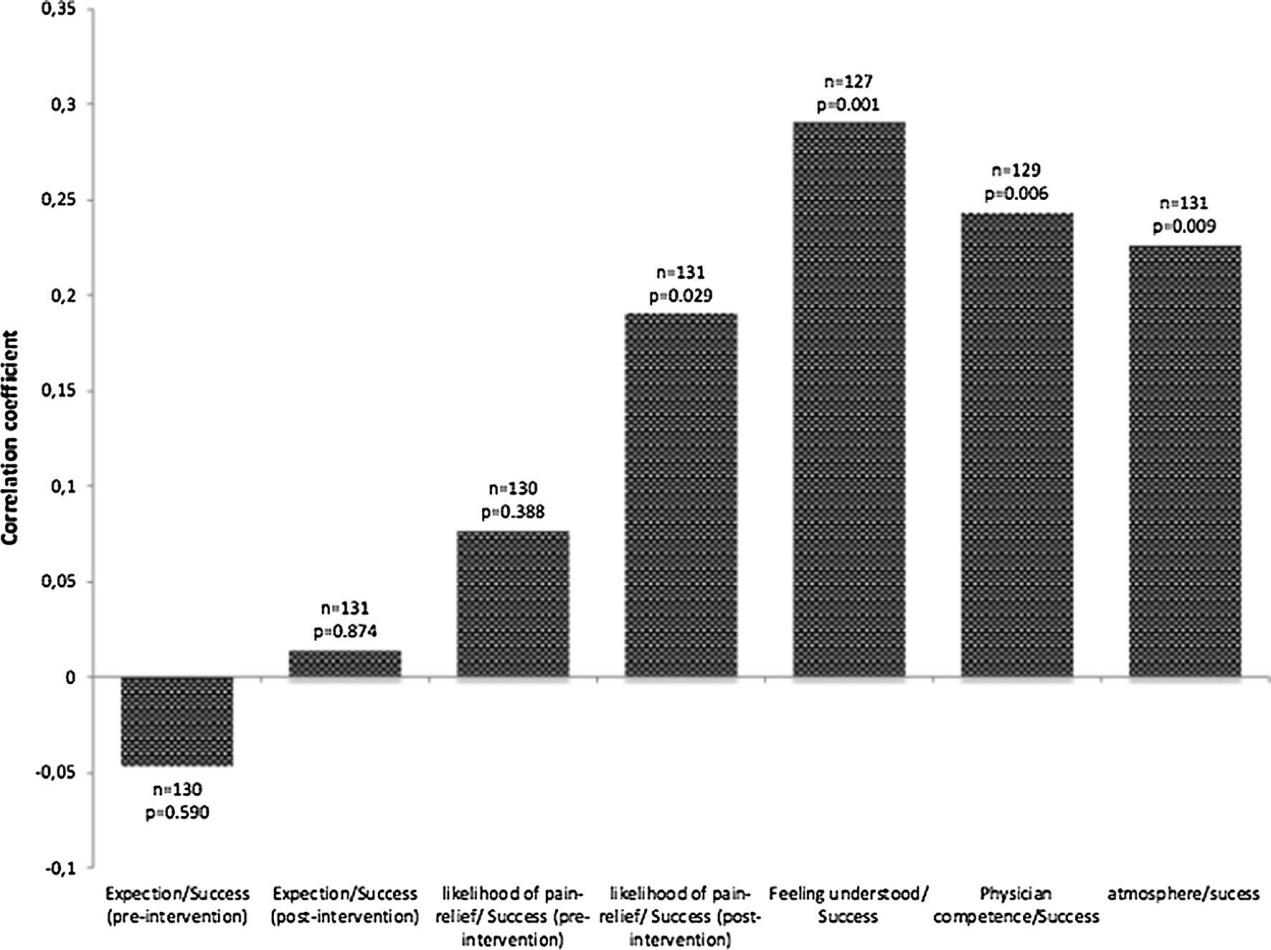
Best results are reached if the patient feels understood by the physician and if the patient recognizes the physician to be competent

The results of the patient questionnaires as well as the patient-demographic dependencies were evaluated with standard statistic approaches. Using Spearman rank correlation, we could show that there exists a positive correlation between the following factors originating from the patient with respect to the outcome:

The outcome of the patient is significantly better if the patient feels understood by the physician concerning his/her condition (correlation coefficient: 0.29, *p* < 0.001). Also, if the patient considers the physician as competent the outcome is significantly better (correlation coefficient: 0.24, *p* < 0.006). However, the most significant observation noted by the psychologist is the atmosphere during the intervention. Using a Spearman rank correlation, the correlation between the pain-relief and the atmosphere was found to be 0.24 (*p* < 0.009) and is of a similar strength as the competency of the physician or if the patient felt understood (Fig. 1).

**Fig. 2:**
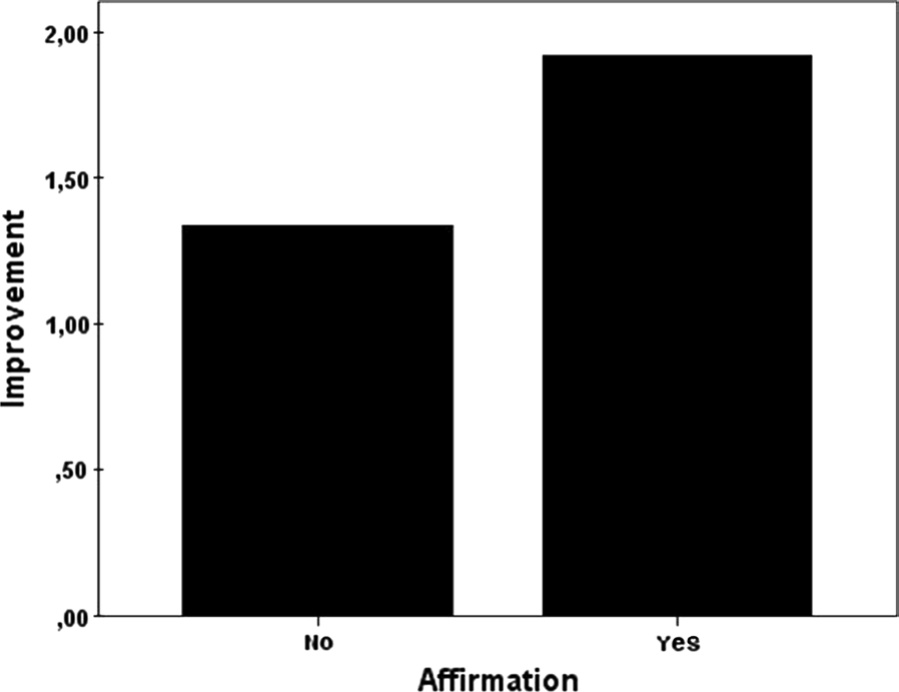
Affirmative statements about a positive outcome lead to a better pain improvement

A positive affirmative statement from the treating physician prior to treatment leads to better outcome for the patient. We could show that if the physician states that the intervention will be successful, there is a greater pain reduction (mean reduction of pain score 1.9) than if such statements are omitted (mean reduction of pain score 1.3) (Fig. 2). Hence positive statements of affirmation are related to a further pain reduction of 0.6 points on the pain scale (t(148) = 2.273, *p* < 0.025).

**Fig. 3:**
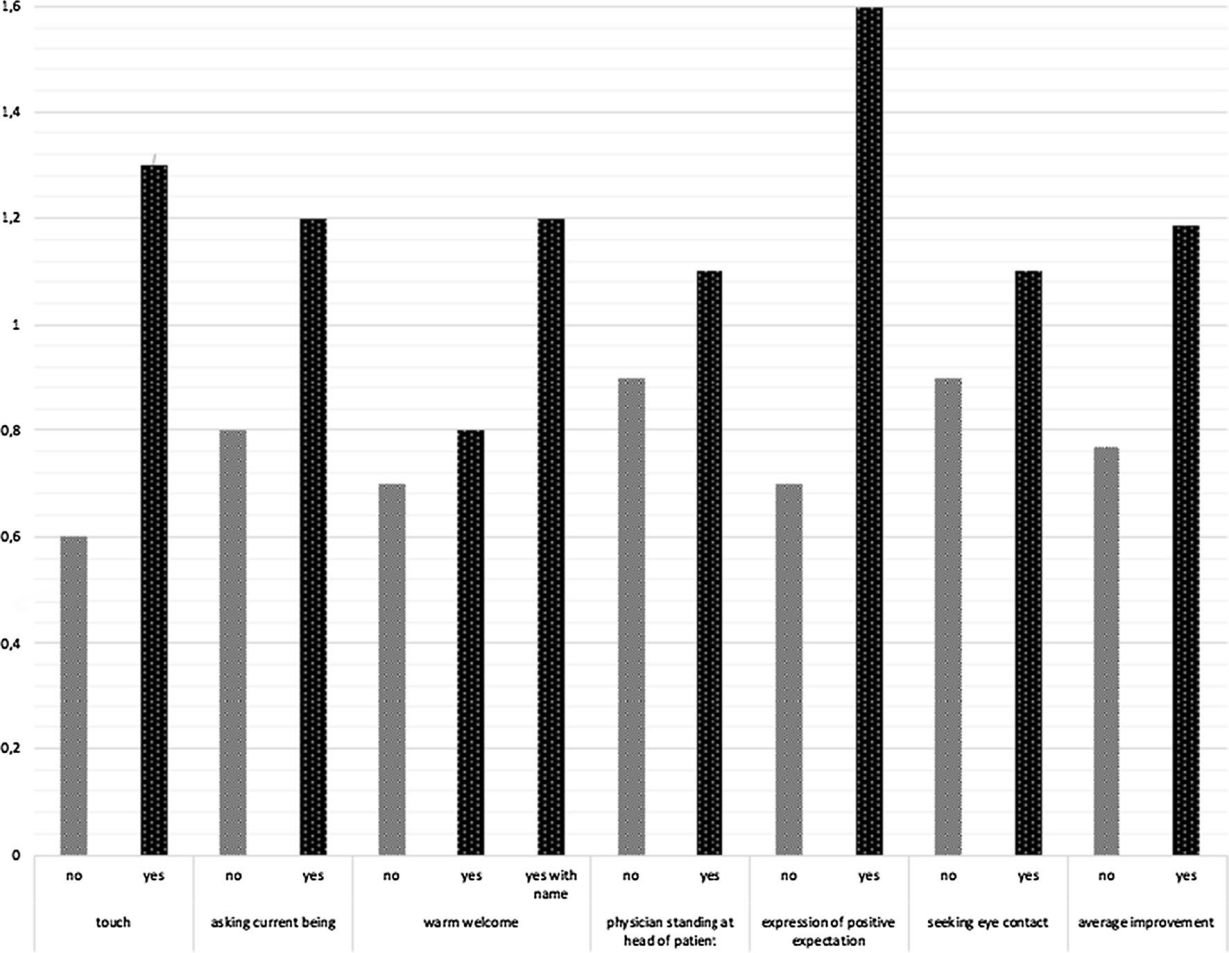
There is a clear but not significant trend. If there is more patient centered interaction between physician and patient during intervention the outcome will be better

**Fig. 4:**
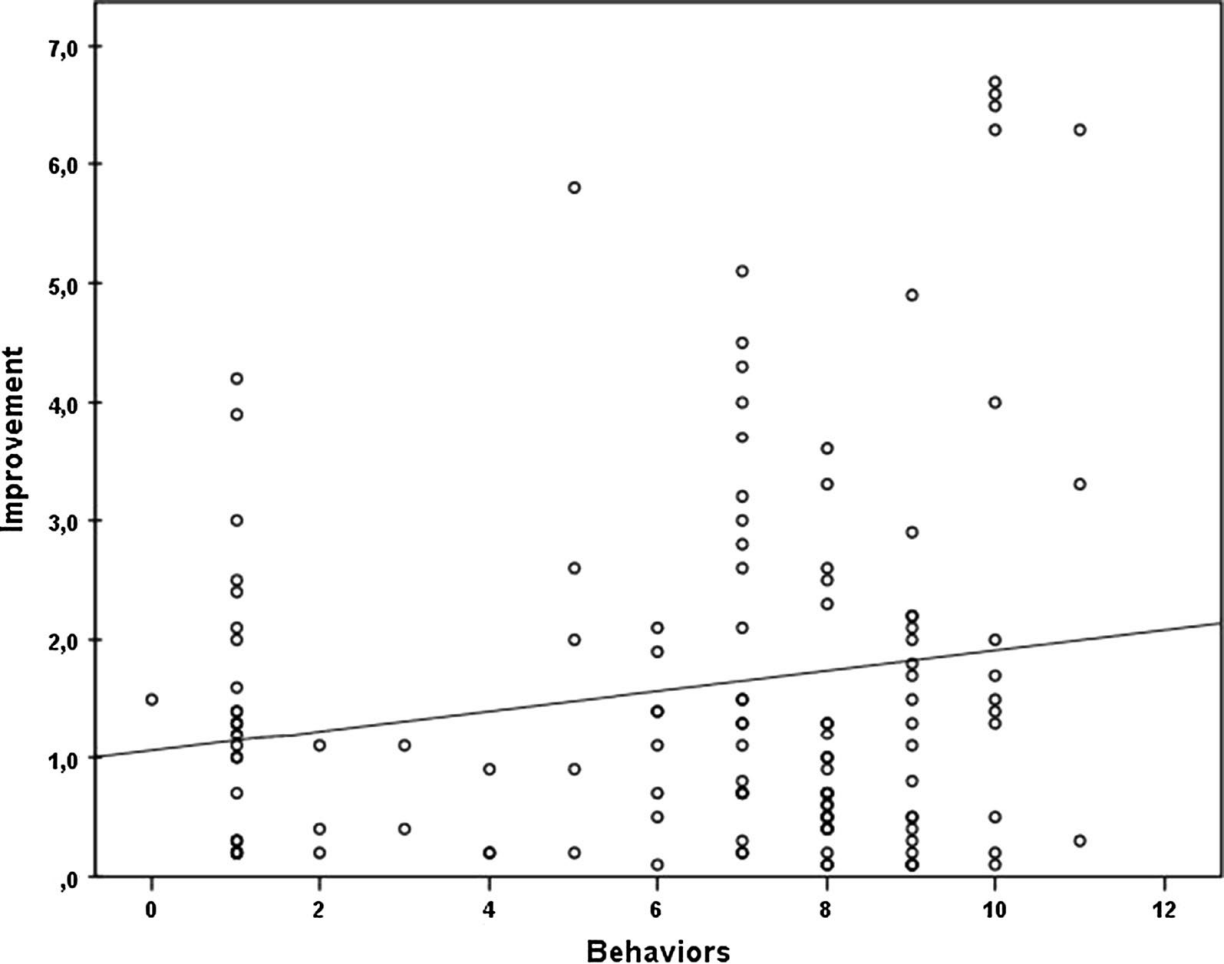
Pain improvement in relation to the total number of specific behaviors of the physician

In addition, there seems to be a small effect on pain reduction from a combination of different specific behaviors of the physician. In particular, the following behaviors were observed: greeting the patient; moving toward the head of and/or seeking eye contact with the patient while the patient lies in the scanner; touching the patient in a reassuring and gentle manner; asking the patient about his/her well-being; explaining the procedure and providing assurance to the patient. In total, the more of these behaviors were displayed, the greater was the reduction on pain ratings measured directly after the intervention (correlation ratio = 0.18, *p* < 0.05) (Figs. 3, 4).

**Fig. 5:**
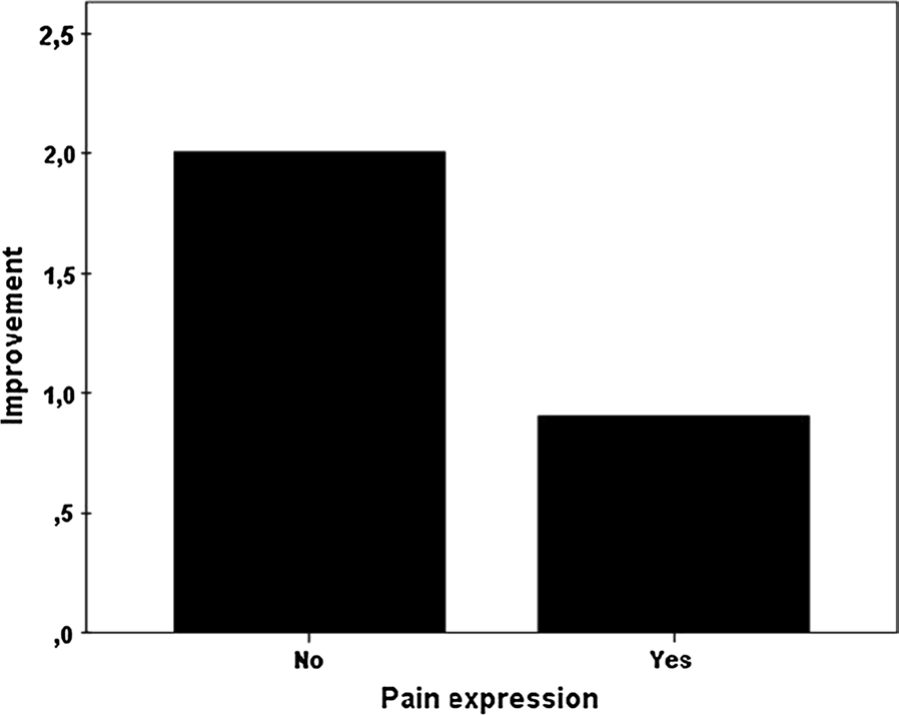
If the patient expresses a feeling of pain during intervention the outcome will be worse

Interestingly, when the patient complains about pain during the intervention the outcome is significantly worse than when the patient expresses no pain (t(49) = 2.606, *p* = 0.018). In the former case the pain reduction is only M = 0.9 points on the pain scale in comparison to M = 2.0 points in the latter case (Fig. 5).

**Fig. 6:**
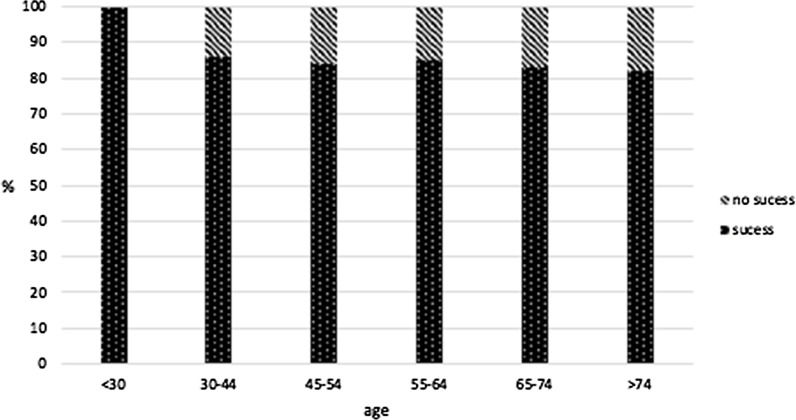
Success rate decreases with increasing patient age

Apart from factors dependent upon the subjective impressions of the patient, we also found that the likelihood of success decreases with increasing patient age, though not significantly (Fig. 6).

Most of the patients showed a benefit from the interventional CT-guided therapy: 86% showed pain relief during and after therapy. The average improvement under therapy was 3.1; the pain score before therapy was 7.2 (3–10) and after therapy 4.1 (0–9). 45% of the patients were also questioned about their pain development in the follow-up; here a consolidation of the success is shown with an average pain score of 3.6 (0–8) and thus an overall improvement of 3.6. However, the follow-up was not always timely, the follow-up time ranged from 4 to 49 weeks.

## Discussion

CT guided pain therapy in specific back pain is an established therapy reaching a significant pain reduction in 63–84% of the treated patients [17–20]. Our study showed comparable results. We observed that 86% of the patients showed significant pain reduction after therapy, but we also found that other independent non-technical factors had an influential effect on the outcome. To our knowledge, no previous study dealt with non-technical factors influencing outcome in CT guided pain therapy. Therefore, we focused on patient-physician interaction before, during and after CT guided therapy.

The occurrence, recognition and processing of back pain involves sensory, cognitive, and affective factors. The impression and rating of pain varies across cultures and individuals [21]. Therefore, the therapy of pain involves different facets of treatment. As one factor, medical treatment serves as a base with a particular dose effect. It is also known from placebo studies that the effect of placebos is dependent on patient-experience, on patient- trust in the treating physician and on the patient mood and temperament [22]. A recent study involves the outcome of chronic spine pain in patients who received interventional and cognitive motivational counseling in combination with analgesic medication. It showed that the combined interventional and cognitive motivational counseling treatment is effective in reducing pain medication [23].

The effects we observed in the current study complement these findings. We showed that interventional pain therapy depends upon the verbal and non-verbal communication of the treating physician with the patient. If the patient felt understood and the atmosphere was supportive, the treating physician was regarded as competent, resulting in a better outcome. If the treating physician expresses a warm welcome combined with a gentle touch and a short statement about the treatment and its expected effects, it has a positive (reducing) impact on pain experience. These actions do not take much time, but significantly improve the outcome.

In the field of CT guided pain therapy these factors have not previously been considered in influencing the therapy outcome, though it is known that side effects from verbal and non-verbal communication exist and influence the variety of therapies and their outcomes [24, 25]. In other fields of medicine there are comparable findings. Back pain is associated with psychosocial factors, fear of movement (kinetopobia/kinesophobia), catastrophizing, and self-efficacy, all of which can be improved by psychological treatment [25].

If in the treatment of specific low back pain only little elements of adequate patient treatment are included the outcome is better. Certainly, the treating radiologist does not have to behave as a psychologist, but when heeding the above-mentioned behaviors, the pain reduction will be better. If the physician gives the patient a conviction of assurance that the treatment will help, the outcome will be better. In psychology this type of therapy is known as framing [24, 26]. It could be shown that to some degree framing also works in medicine. Our results support this thesis.

There is literary evidence that patient-therapist interaction influences the outcome in patients with low back pain [27]. The study compared the outcome of patients with low back pain and divided the patient sample into two groups. A family physician saw one group, and the other group was seen and treated by a chiropractor. In the group treated by the physician there was one consultation and prescription of anti-inflammatory drugs. In the chiropractic group there were 4 visits with a longer duration. The outcome of the chiropractic patients was better with respect to pain reduction as well as to satisfaction with the therapy. This outcome seems to not only depend on the different therapy forms but also on the paid attention and on the satisfaction with more patient centered treatment [27]. Corresponding to this we found a positive correlation between feeling understood by the physician as well as having the impression of a competent physician.

The field of empathy and its affection on the patient–physician relationship is of general interest and often hard to measure. Up to date there is evidence in different fields of medicine and in psychology that a trustful and well defined patient–physician relationship provides a better patient outcome [28]. Also, multi-modality pain therapy offers more intervention time per patient by different forms of therapy. Within one week patients experience psychologically-guided group conversation, psychological lectures, physical therapy, and various medical consultations [29, 30]. Moreover, in the field of multimodal pain management the injection therapy is one part of the therapeutic concept, but in addition physical therapy and sports therapy as well as instruction in progressive relaxation techniques are given. In addition, an individual psychological interview, psychological group therapy, and psychological lectures are all carried out [30]. Bendigs et al. reported a significant pain score decrease in patients with low back pain treated by multimodal pain management therapy and state that a minimal invasive therapy leads to success on most of the patients with specific low back pain [30]. It could be hypothesized that multimodal pain management therapy shows an improvement of pain because the patient is a main focus for treatment and the interest of various therapists for a comparable length of time [30]. This empathy works in distinct ways; multimodal pain management shows a clear focus on the patient and his/her complaints, but it is expensive and time-consuming [2]. Because financial resources in health systems are limited, it would be favorable to isolate dimensions that help to gain a good outcome with relatively low effort. Our study shows that, concordant to the literature, non-verbal communication could be as equally important as verbal communication [28]. Taking the possible patient centered forms of physician behaviors into consideration, there is a clear trend to reach better pain reduction if there is friendly and competent interaction with the patient [28].

Our study shows in some respects that the effectiveness of the therapy depends on the treating physician. The most successful physician is the one with the most experience. However, when all five attending physicians are considered together, we could not prove that the physician's experience is positively correlated with the patient outcome. We also could not show a significant correlation for the age of the physician and gender. The purely technical factors can be excluded to a large extent by the study design we have chosen, as the CT controlled procedure ensures a comparable drug distribution in all cases included in the study. In addition to experience, a number of other factors are also discussed in the literature which are required to achieve an optimal result for the patient [31, 32].

Our study has limitations. The number of included patients and observed examinations is comparable small, therefore our small sample size limits the presented study. Only 45% of the patients were willing to participate in the final survey a few weeks after the intervention. Even if the assumed trend towards improvement of pain symptoms was confirmed in these patients, the low number of response considerably limits the statement on long-term success. The number of participating physicians was too small to identify clear personal characteristics that could influence the success of the treatment. There is strong evidence to support that verbal and non-verbal interaction between physician and patient has a significant effect on patient outcome, but we could not trace which behavior and rituals are best to achieve a patient’s optimal outcome. The study was not video analyzed, so there could be details in the interaction between physician and patient that were not recognized and reported by the observing psychologist.

## Conclusion

Outcome of CT guided therapy is significantly correlated to the treating physician’s behavior. The pain reduction is significantly better if the atmosphere is friendly and patient centered, if the physician is competent in the view of the patient, and is understanding of the patients’ complaints. Additionally, framing before therapy leads to better results.

## Data Availability

All data generated or analyzed during this study are included in this published article.
